# Cow-calf contact: a single-herd observational study of AMS yield during the first 100 days in milk

**DOI:** 10.1186/s13028-024-00757-7

**Published:** 2024-07-17

**Authors:** Henrik Hanssen, Hanne Amundsen, Julie Føske Johnsen

**Affiliations:** 1 Department of Animal and Aquacultural Sciences, Faculty of Biosciences, Norwegian University of Life Sciences, Oluf Thesens vei 6, Ås, 1433 Norway; 2 Present address: God på Gris, Dalveien 82, Sandnessjøen, 8804 Norway; 3 Søndre Vivelstadsvea, Åsbygdsveien 876, Rena, 2450 Norway; 4 Department of Animal Health, Welfare and Food Safety, Section for Terrestrial Animal Health and Welfare, Norwegian Veterinary Institute, Elizabeth Stephansens vei 1, Ås, 1433 Norway

**Keywords:** Calf welfare, Norwegian red breed, Performance, Suckling

## Abstract

An increasing number of dairy farmers plan to implement cow-calf contact (CCC) in their herd which necessitates descriptions of the cows` performance in different systems. The aim of the study was to describe (1) Automatic milking system (AMS) milk yield of cows in a CCC system during the first 100 days in milk (DIM) and (2) AMS milk yield before and after cow-calf separation. In a prospective study at a commercial Norwegian dairy farm, we included all calvings from Norwegian Red cows between January 2019 to April 2020. After calving, cow-calf pairs stayed in an individual calving pen during the first 5–6 d before they were moved to the loose housing unit with the remaining herd. Calves had whole-day (24 h/d) and full physical contact to the cows. Cows were milked in an AMS. From 14 individual cows of which one cow calved twice during the study period, we collected daily AMS yields from 15 different lactations, with parities ranging from 1 (*n* = 6), 2 (*n* = 5) and 3 (*n* = 4). Due to the sample size and structure of the data set, we only calculated descriptive statistics from DIM 7-100. All data is shown separately for primiparous and multiparous cows. Mean (± SD) calf age at (fence-line) separation was 52 d ± 14.8 beyond which suckling was prevented. Our data indicates great individual variation in the AMS milk yield. Prior to separation, primiparous cows` AMS yields ranged from 11.0 to 25.9 kg/d while that of multiparous cows ranged from 4.8 to 28.8 kg/d. Once calves were no longer allowed to suckle, the yield increased gradually. During the week after separation, AMS yields ranged from 17.3 to 30.4 kg/d for primiparous cows and 8.7 to 41.8 kg/d for multiparous cows and these yields increased in DIM 93–100 (26.5 to 34.3 and 20.6 to 38.3 kg/d respectively). This study is limited by a low sample size from a single-herd but may provide useful descriptions of AMS milk yield in a whole-day, full contact CCC system during the first 100 days of lactation.

## Findings

Most commonly, dairy calves are separated from their dam shortly after birth. However, 3% of Norwegian dairy farmers now practice cow-calf-contact [CCC, 1] during the first weeks of the lactation [2]. In [2], the authors found that as many as 15% of the farmers plan to implement CCC on their farm. In CCC systems, cow-calf pairs have different degrees of physical contact (full or partial) while the duration of daily contact may vary from whole-day CCC to part-day CCC [1]. EFSA recently stated that CCC should be progressively implemented as more knowledge becomes available [3]. Documented assets of CCC for the calf are related to e.g., a high pre-weaning growth [4], a positive or neutral effect on health [5] and the possibility to express natural social and feeding behaviours [6, 7]. Given the pivotal importance of a stable income from the dairies, farmers considering to change from artificial calf rearing to CCC need knowledge on the expected performance [5]. In CCC systems, the cows’ total milk production constitutes the suckled milk (often not quantified) and the machine milk yield [8]. Many Norwegian farmers practicing CCC use Automatic Milking Systems (AMS) [9] which necessitates descriptions of AMS performance specifically. The aim of the study was to describe (1) AMS milk yield of cows in a CCC system during the first 100 days in milk (DIM) of the lactation and (2) AMS milk yield before and after cow-calf separation.

This study took place at a commercial dairy herd in Rena, Norway practicing dam-calf contact since 2018, i.e., the calf had contact to its own dam [1]. This herd had 15 lactating dairy cows. The breed was mainly Norwegian Red (*n* = 14) and Jersey (*n* = 1). The dairy herd manager (HA) was willing to participate in an observational study and share data.

All Norwegian Red cows calving during the study period (January 2019 to April 2020) were eligible for inclusion. Exclusion criteria were failure of established suckling or bonding, calving outside the calving pen or a difficult parturition resulting in clinical signs of ill-health of cow or calf. Calving took place in an individual calving pen. The calving pens were bedded with straw (until the calf was born) or sawdust. For the first 5–6 d after birth, pairs were housed in the calving pens. During these days, cows were taken to milking in the AMS (Lely A4, Cornelis van der Lelylaan 1, 3147 Pb Maassluis, The Netherlands) twice per day. Cow-calf pairs were then moved to the main area for lactating cows. This area consisted of two loose-housing areas: the fresh-cow pen (68 m^2^) and the main pen for lactating cows (174 m^2^). At d 5–6, a cow-calf pair was moved to the fresh cow pen (five lying cubicles and five feeding slots) for one week. Subsequently, they were moved to the main pen with other lactating cows and their calves. This area had 22 rubber matted and saw-dust bedded lying cubicles and 30 feeding slots. Manure was removed from the closed floor with a manure robot collector (Lely Discovery 120 Collector, Cornelis van der Lelylaan 1, 3147 Maassluis, The Netherlands) and deposited in a designated area with perforated floor. Calves had whole-day (24 h/d) and full physical contact to their cows. In this period, calves could suckle the dam (or potentially other cows) freely. All resources (lying cubicles and the cows feed) were shared between cows and calves.

The event of separation refers to prevention of cow-calf suckling. This occurred at mean (± SD) calf age = DIM 52 d ± 14.8. From this age, cows were housed in the fresh cow area while the calves were moved to an adjacent calving pen, entailing that calves had fence-line contact (i.e., physical and visual contact, but not suckling) to their dams for 3–4 d. Because calves were always separated in pairs, age at separation varied according to calving date. At separation, all calves were trained to drink milk from a teat bottle (twice per d, feeding to satiety). Following fence-line separation, cows were moved to the main lactating herd where visual, but not physical contact to the calves was maintained. At 12 weeks, calves were weaned off milk.

For each individual cow-calf pair, we collected data on cow parity, date of birth, date cow and calf were moved from the calving pen and first day of separation. Daily AMS milk yield and DIM of the individual cow was collected with Lely software and stored in a local database.

The outcome in this study was individual cows’ daily AMS milk yield which was obtained by adding AMS milk yields from each AMS visit. Due to the sample size and structure of the data set, no statistical analyses were performed. Mean daily AMS milk yield was calculated per 24 h using 3-d rolling averages. Descriptive statistics and graphs were produced using Stata SE/16 (Stata Corp., College Station, TX, USA). The suckled milk yield was not quantified. The study used data from DIM 7-100. All data is shown separately for primiparous and multiparous cows. Individual variation in daily AMS milk yield is presented graphically from DIM 7 to 100 in line graphs. Bar graphs showing mean and 95% confidence intervals are used to show variation in daily AMS milk yield in response to separation of cow and calf. In addition, descriptive statistics (mean, standard deviation, range) were calculated for each of the two lactation periods: (1) *the week prior to separation* denoting days − 7 to -1 relative to the first day of separation (= day 0) and (2) *DIM 93–100*.

Due to failure of established suckling or birth outside the individual calving pens, five cows were excluded from the dataset. One cow calved twice during the study period. Final sample size therefore included data from 15 lactations (from 14 unique cows) with parities ranging from 1 (*n* = 6), 2 (*n* = 5) and 3 (*n* = 4). One cow was treated for mastitis, the data was excluded for the two days before and after treatment of the mastitis (8d are missing). The final dataset contained 1401 observations (= milking days), with an average of 93 observations per cow (minimum 85 and maximum 94).

**Fig. 1:**
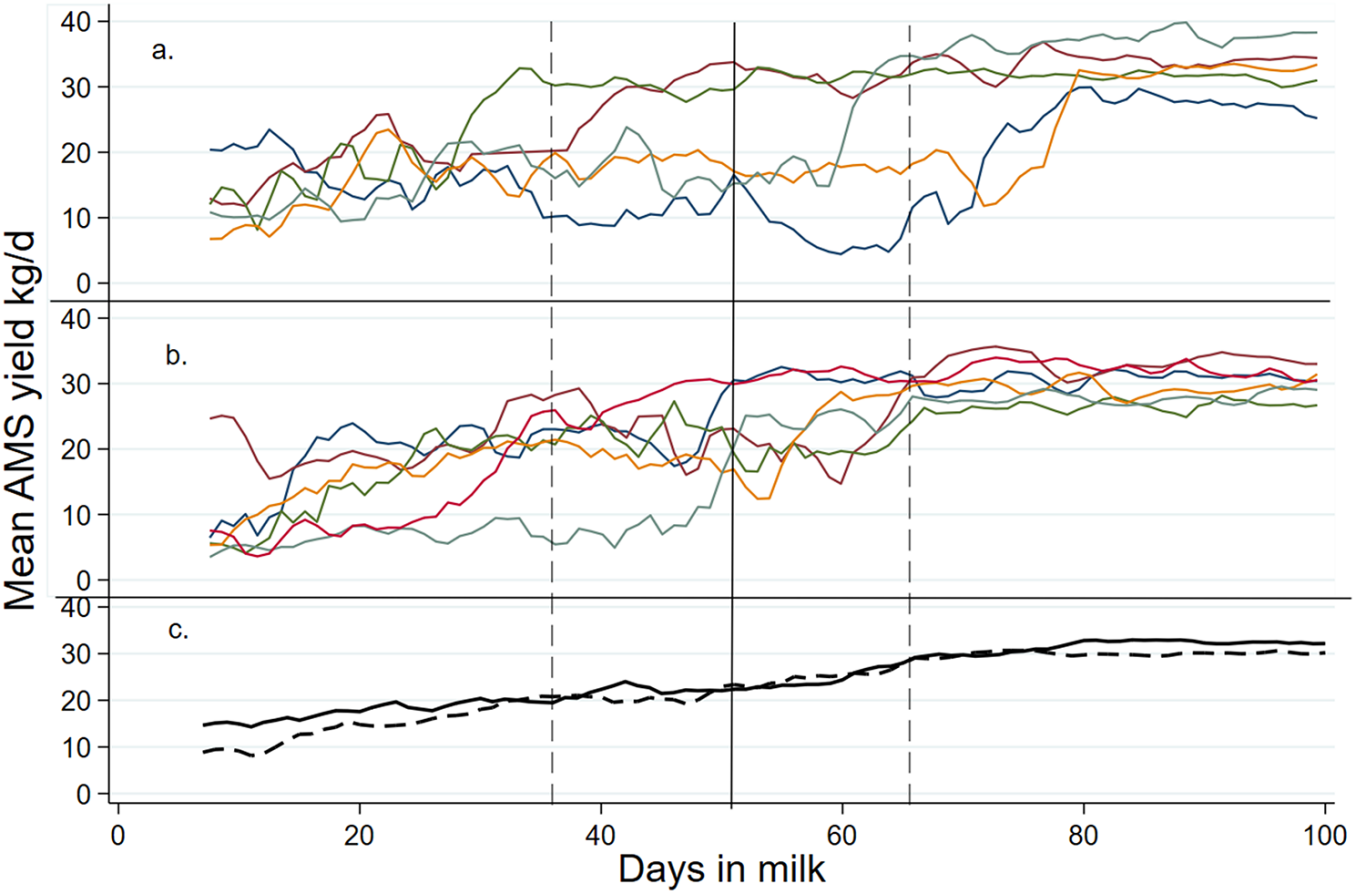
, **a**-**c**. Cow milk yields in a cow-calf contact (CCC) system over days in milk (DIM) 7-100. Mean daily AMS milk yield for lactations (*n* = 15) from 14 different cows suckling their calf in a whole day and full contact CCC-system over days in milk 7 to 100. Calves were fence-line separated from their cows at mean (± SD) age of 52 days (indicated with a black vertical line) ± 14 d (each upper and lower SD is indicated with vertical dashed lines). Data is shown separately for individual primiparous cows (**a**) and multiparous cows (**b**) while (**c**) shows mean daily AMS milk yields for primiparous (dashed line) and multiparous cows (solid line)

Daily AMS milk yield varied substantially with individual cows, especially during the early suckling period (Fig. 1). During the week prior to separation, primiparous and multiparous cows produced 20.0 ± 3.19 kg milk/d and 16.8 ± 5.21 kg milk/d, respectively (Table 1). Our results indicate that one multiparous cow had a lower yield (4.8 kg/d) and a different lactation curve than that of the other cows. With limited sample size, this clearly affects the mean yield. This finding is in line with two other recent Norwegian studies; one studying a cow-directed CCC system, demonstrating mean AMS yield during the suckling period 13.2 ± 7.8 kg, 10] and one study on CCC cows kept on pasture (mean daily machine milk yield during the suckling period 10.8 ± 5.5 kg, [11]). In comparison, Norwegian Red cows with no CCC were found to produce 27.9 ± 8.65 kg. [12]. This variation may be linked to the calf since milk intake of artificially reared calves fed high allowances varies with individual calves, especially in early life (min-max milk intake during the first week of life = 3–11 L/d, [13]. Another factor that may explain the individual variation may be decreased milk secretion linked to incomplete evacuation of milk from the udder as suggested by Barth et al. [14]. Finally, some cows are more prone to nurse «alien» calves in the herd [15, 16]. We did not record cross-suckling in this study, and can therefore not exclude that this behaviour may have influenced the outcome.

**Table 1:** Milk yield of Norwegian red dairy cows in a cow-calf contact system

Lactation	Period	*N* (lactations)	AMS milk yield^2^, kg/d		
			Mean (± SD)	Min	Max
1	The week prior to separation^1^	6	20.0 (3.19)	11.0	25.9
	DIM 93–100		30.1 (2.22)	26.5	34.3
> 1	The week prior to separation	9	16.8 (5.21)	4.8	28.8
	DIM 93–100		32.3 (4.80)	20.6	38.3

^1^ Calves were fence−line separated from their cows at the mean (± SD) age of 52 ± 14.8 days. In the category *week prior to separation*, one lactation had missing observations due to mastitis (data missing on d −2 to 6 relative to separation)

^2^ Rolling average over 3d

Descriptive statistics of daily automatic milking system (AMS) milk yields of 14 cows and 15 lactations in a cow-calf contact (CCC) herd prospectively studied over 7-100 days in milk (DIM). Calves suckled the cows for 6–8 weeks in a whole-day, full contact CCC system. Data is shown relative to separation (= day 0) which entails that calves are fence-line separated from the dams (3–4 d) prior to total separation (i.e. prevention of any physical contact)

**Fig. 2:**
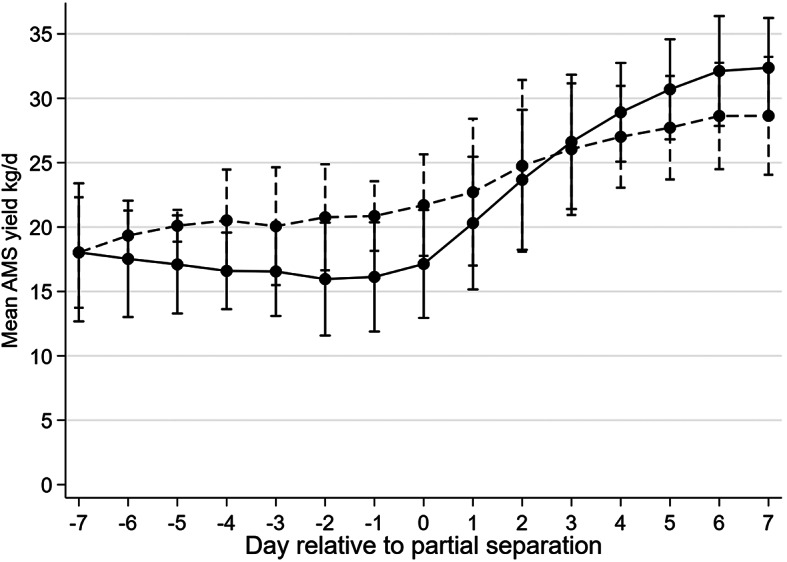
Milk yield before and after cow-calf separation. The figure shows how mean daily AMS milk yield for cows (*n* = 14) suckling their calf in a whole day and full contact CCC-system varied in response to (fence-line) separation (= day 0). Whiskers represent 95% confidence intervals of the mean. Data is shown separately for primiparous cows (dashed line) and multiparous cows (solid line)

AMS milk yield numerically increased after separation (i.e., d 0), likely because calves could no longer suckle (Fig. 2). Multiparous cows’ yield intercepted that of primiparous cows, indicating a steeper increase in AMS milk yield after separation, compared to primiparous cows which had a greater individual variation post separation. Compared to the week prior to separation primiparous and multiparous cows increased mean yields with 10.1 and 15.5 kg, respectively. Premature (i.e., before «natural» weaning age of 9–12 months, [17]) separation of the calf from the cow elicits strong behavioural responses that likely also affects milk secretion [18, 19]. Individual cows’ vocal and behavioural response to separation is highly variable [19] and future research may investigate its association with how soon cows’ AMS milk yield increases in response to separation from the calf. Also, since a suckling calf often evacuates milk unevenly from the udder an effective synergy with quarter level-milking may be present in AMS systems. However, future research should investigate if improved milking management (e.g. frequent milking, enhanced pre-stimulation etc.) can mitigate individual variation in AMS yield during separation.

This study is limited by a small sample size from a single herd but may provide useful descriptions of AMS milk yield in a whole-day, full contact CCC system during the first 100 DIM. AMS yields of nursing NRF cows displayed significant individual variation and once calves were no longer allowed to suckle, the steepness of the yield increase may be related to parity.

## Data Availability

The datasets used and analysed during the current study are available from the corresponding author on reasonable request.

## Declarations

### Prior publication

Data have not been published previously.
